# Supporting parents and healthy behaviours through parent-child meetings – a qualitative study in the Netherlands

**DOI:** 10.1186/s12889-021-11248-z

**Published:** 2021-06-18

**Authors:** Gülcan Bektas, Femke Boelsma, Carline L. Wesdorp, Jacob C. Seidell, Vivianne E. Baur, S. Coosje Dijkstra

**Affiliations:** 1 Department of Health Sciences, Faculty of Science, Vrije Universiteit Amsterdam, Amsterdam Public Health Research Institute, De Boelelaan 1085, 1081 HV Amsterdam, the Netherlands; 2 Department of Care Ethics, University of Humanistic Studies, Kromme Nieuwegracht 29, 3512 HD Utrecht, the Netherlands

**Keywords:** Early childhood, Parenting, Peer support, Playgroups, Community support group, Health education, Health promotion

## Abstract

**Background:**

The first 2 years of a child’s life have been found to be crucial to healthy growth and development. Parent support groups can help parents to promote health-related behaviours during this crucial period. The aim of this study was to explore the experiences of parents who participated in a parent support group (Parent-Child Meetings) which promoted health-related behaviours of their children, and to determine whether and how these meetings supported them in promoting these behaviours.

**Methods:**

We used a qualitative study design. The parent support group consisted of weekly Parent-Child Meetings organized in a multi-ethnic, relatively low-income neighbourhood in Amsterdam, the Netherlands. Data on the experiences of parents was collected through participatory observations, informal conversations (*n* = 30 sessions) and semi-structured interviews (*n* = 13) between April 2019 and March 2020. The data was analysed using thematic content analysis.

**Results:**

Parents indicated that they experienced the parent-child meetings as enjoyable and as providing them with socio-emotional support. They reported that the meetings increased their parenting knowledge, skills and practices regarding healthy behaviours of their children and that they used this knowledge in their daily lives. They also appreciated the practical information and advice provided by experts in the meetings. Parents indicated that the positive attitude of the experts was crucial in accepting and adopting their advice. Additionally, parents valued the interactive and hands-on workshops, which integrated health-related behaviours and active play with children, as it enabled them to learn while they played with their children.

**Conclusion:**

This study indicated that parent-child meetings contributed to enhancing parental knowledge, skills and practices regarding healthy behaviours of their children. This could potentially benefit the health of children during the first 2 years of their lives. In particular, the peer support of other parents, the hands-on workshops, and the concrete advice and information provided in an informal setting were highly valued by parents. Future parent support groups could use these findings to improve their meetings or to start meetings that better suit the needs of parents with young children.

**Supplementary Information:**

The online version contains supplementary material available at 10.1186/s12889-021-11248-z.

## Background

The first 2 years of a child’s life provide a crucial window of opportunity to support childhood development and long-term health [1–3]. Health-related behaviours, including diet, sleep, physical activity and several associated determinants of good health are established during this critical period and are known to impact later childhood and adulthood [4, 5]. Inequalities in health and related behaviours also occur in early childhood and are closely linked to the socioeconomic position (SEP) and ethnicity of parents [6–8]. Various studies have shown that children of families with a lower SEP have a higher prevalence of childhood obesity due to less healthy dietary patterns or low levels of physical activity compared to children of families with a higher SEP [9–12].

Parents play a key role in the support of health-related behaviours, especially during the early period of life when children depend on their parents to a large extent for a range of needs [13, 14]. These health-related parental practices relate to their choice of certain behaviours, such as the use of schedules or limits concerning unhealthy foods, active involvement in children’s activities, or setting rules around screen and/or sleep time [15, 16]. Some of these practices have a far-reaching impact on the health-related behaviours of children, such as food choices and dietary patterns, interest in physical activity and length of screen time [15, 17–20]. Therefore, it is important that parents are aware of the relationship between the establishment of healthy behaviours in early childhood and their long-term health consequences.

However, several studies have shown that parents of young children are uncertain or lack knowledge about a healthy diet, daily physical activity, adequate sleep, and limited screen time for their children, and that they experience problems or are unable to facilitate a healthy environment for their child [21–25]. Moreover, the transition to parenthood is a major adjustment period and is known to be a time where the need for support is increased, since it comes with profound life changes and considerable stress [26–28]. Many parents thus appreciate support from family members or their social network in acquiring parenting skills [26, 29]. Additionally, parents appear to have a desire to meet other parents (peers) to learn from each other and receive peer support, and to gain information and practical tips regarding parenting through real-life experiences [21].

Parent support groups can also engage with parents by providing some form of informal support that allows parents to share similar experiences on parenting in a setting that is safe and non-judgmental [30–32]. Parent support groups, which aim to bring parents together, are already found in many countries, albeit under different names and with different purposes and formats. It has been shown that these groups provide social support, increase parenting confidence and improve parent-child relationships [33–36]. In addition, there is also evidence that parent support groups may play an important role in facilitating and supporting effective parenting practices concerning health-related behaviours [31, 37]. However, most interventions in playgroup settings focus on promoting one healthy behaviour, such as healthy food or physical activity, and there are limited studies in these kinds of settings that target multiple health-related behaviour domains [35, 38]. However, most interventions in playgroup settings focus on promoting one healthy behaviour, such as healthy food or physical activity, and there are limited studies that target multiple health-related behaviour domains together [35, 39–42]. In addition, several studies have shown that parental engagement and strong collaboration between parents and (health) professionals are important factors for successful health promotion programs for parents and their children [35, 40–43]. However, there are still limited studies of successful parenting programs in which facilitators and parents worked in partnership to promote healthy behaviour in children during the earliest life stages, and gaps remain in our knowledge of the results of parenting programmes which are developed in active collaboration with parents, particularly in multi-ethnic lower socioeconomic communities [21, 41, 43].

Based on these findings, we started facilitating weekly parent-child meetings, which is designed in collaboration with parents, in the context of the ‘Food4Smiles’ research project. In addition to social interaction between parents and children, the parent-child meetings focused on multiple domains of health-related behaviour, which aimed to improve parental practices, skills and confidence concerning healthy behaviour. During the meetings, several health professionals were invited to share information with the group about various health-related behaviours (e.g. healthy food, physical activity, adequate sleep, relaxation, oral health).

The aim of this study is to explore the experiences of parents who participated in these parent-child meetings and to investigate *whether* and *how* these meetings supported them in promoting health-related behaviours in their children. This study took place in a relatively low-income and multi-ethnic neighbourhood in Amsterdam, the Netherlands.

## Materials and methods

### Study design

This study is part of the ‘Food4Smiles’ research project, which aims to promote the healthy growth and development of children during the first thousand days of life, focusing on a relatively low-income and multi-ethnic neighbourhood in Amsterdam, the Netherlands. Because the purpose of this study was to explore participants’ perceptions and experiences, an experiential qualitative descriptive design was chosen [44], based on participatory observations, formal interviews and informal conversations [45, 46]. This design allowed a rich and in-depth understanding of participants’ experiences, while acknowledging the role of both the participants’ understanding and the researchers’ interpretations in the production of knowledge [45].

### Parent-child meetings

In April 2019, weekly parent-child meetings were initiated and organized in collaboration with a ‘city farm’ located in the neighbourhood. The content and the structure of the meetings were designed in close collaboration with parents who had participated in our previous explorative study [21]. The aim of these meetings was to organize activities to promote health-related behaviours during the first 2 years of the children’s lives, through social interaction, practice and observational learning in a safe and informal setting. The groups were facilitated by a community development worker, who was assisted by the principal researcher between the period April 2019 and March 2020. The community worker and the principal researcher also lived in the neighbourhood.

Each parent-child meeting lasted 2 h and started with a walk-in of 30 min, which was followed by a check-in moment that involved singing children’s songs together. Subsequently, the facilitators of the meetings organized different activities, for example painting or different craft activities. There were also regular informative workshops during the meetings, which addressed several health-related behaviours (e.g. healthy food, physical activity, adequate sleep, oral health and relaxation). These informative workshops (e.g. baby massage, preparing and tasting healthy baby snacks, tooth brushing, toddler yoga or gym) were led by health professionals (e.g. paediatrician, dentist or paediatric physiotherapist) and/or health ambassadors. Health ambassadors are local volunteers who are appointed by the municipal health service to work in their own neighbourhood after completing training in health and lifestyle-related topics. The informative workshops were all designed differently, for example some workshops were meant for parents to discuss a health-related topic and exchange information by sharing experiences and did not involve the children. Other workshops were designed as an interactive and integrated workshop, where both parents and children were involved in the activities.

The parent-child meeting groups operated on a ‘drop in’ basis, which means that there was no requirement to sign up for the group, and participants could attend the meetings regularly or occasionally. Each week, between 15 to 20 parents attended the meetings with their children. The composition of the groups was diverse in terms of the sex, age, ethnic background and education level of the individual attendees. Fathers, grandmothers and aunts occasionally attended the meetings, but the majority of the participants were mothers.

### Recruitment

Parents were informed about and invited to the parent-child meetings by other parents who had participated in our explorative study [21]. These parents collaborated in the design and set-up of the parent-child meetings and handed out flyers to other parents in their own neighbourhood. Parents were also informed and invited to the parent-child meetings through social media (Facebook, Instagram), and using flyers and posters which were placed in various health-care settings, playgroups, community centres, libraries and local supermarkets. The parent-child meetings were also promoted by the attending parents and attracted other parents from the neighbourhood. Recruitment targeted parents or caregivers with infants aged 0–2 years, and they were allowed to bring along other siblings up to the age of 4 years.

In total, 20 participants who regularly attended the parent-child meetings were approached for an interview and 13 agreed to participate. With the agreement of the participants, 9 interviews were conducted at the participant’s home or at the city farm, while 4 interviews were conducted by telephone due to COVID-19 circumstances.

### Consent and ethical considerations

In the first parent-child meeting, an introduction to the researcher (GB) took place, as well as an introduction to the project. All participants were aware of the goal of the study and the role of the researcher. Subsequently, the researcher introduced herself and explained the goal of the study to each new participant individually. All participants provided oral consent for the participatory observations and informal conversations and the anonymous reporting of the observations and findings.

All participants who took part in the formal interviews received a letter informing them about the study, and all of them provided written or recorded informed consent for participating in the study. The recorded consent was obtained by reading the informed consent form and asking for permission to involve participants in the research, which is recorded. The participants consented to take part in the study. The interviews were transcribed verbatim and anonymized by removing identifying characteristics from the data. This study was approved by the Medical Ethical Committee of Amsterdam UMC (VUmc location).

### Data collection procedures

Between April 2019 and March 2020, participatory observations and informal conversations occurred during 30 meetings lasting 2 h each (total 60 h). The participatory observations were used to observe what participants did during the parent-child meetings and provided opportunities to initiate informal conversations with the participants. This allowed the researcher (GB) to build long-term relationships with the participants, which led to more detailed observations, greater attention to and a more insightful investigation of participants’ behaviour and social interactions [47]. Field notes on the observations and subsequent informal conversations were made after each observation. The observation notes included the activities of participants and information about the context, interactions and conversations with and/or between participants. The informal conversations were open-ended and allowed the researcher to learn about individual participant’s thoughts on the activities during the meeting and personal background information about their home situation and cultural/traditional beliefs or practices regarding child rearing.

Additionally, between February 2020 and March 2020, formal interviews were conducted with 13 participants to obtain more in-depth information about their experiences of the parent-child meetings. The interview guide was designed using themes from the literature, the participatory observations and the informal conversations, which made it possible to discuss topics in more depth during the interview. This also allowed us to verify and confirm the observations and findings from the participatory observations and informal conversations and to enrich the information about the experiences of participants in the parent-child meetings. The interview guide included topics such as participants’ experiences of the informative workshops, how they contributed to health-related behaviours in their children, changes in daily practices and the health-related behaviour of their children, and the social support experienced (see Additional file 1). The interviews varied in length from 25 to 45 min. Every interview ended with additional questions about the age of the participant, the mother’s and father’s highest attained level of education, ethnic background, employment status and number and age of children. Educational level was classified as no education/primary education (low); lower secondary education/higher secondary education (middle); higher vocational college/university (high). Ethnic background was established with reference to the country of birth: either the respondent’s own country of birth or the country of birth of one of their parents. The interviews and informal conversations were all conducted in Dutch.

### Data analysis

A thematic content analysis using the software programme ATLAS.ti [48] explored the observation notes and formal interviews, with the aim of being as open as possible to the experiences and perspectives of the respondents. The observation logs included the activities and information for all participants who attended the parent-child meetings. The formal interviews (*n* = 13) were coded and checked by two researchers (GB and FB) independently to increase reliability and ensure uniform coding. They both identified recurring themes in the interviews and coded data into concept categories. While consensus was reached on most topics, some topics were further explored to find consensus. After the axial coding process, the categories were abstracted into themes that reflected the variety of experiences of participants involved in the parent-child meetings.

## Results

The parent-child meetings attracted participants from diverse ethnic backgrounds, and the majority of the participants lived in the study district. We estimated that most participants had a Moroccan, Dutch or Turkish background. There were a few regular participants who came from other neighbourhoods of Amsterdam or even other cities in the vicinity of Amsterdam. Almost all participants who attended the parent-child meetings and participated in the interviews were mothers, and therefore we will use the term ‘mothers’ to describe our participants below. Table 1 presents the characteristics of the mothers who took part in the formal interviews.

**Table 1:** Characteristics of the mothers who participated in the formal interviews (total *N* = 13)

**Age in years, mean** ***(range)***	31.2 *(28–39)*
**Education level mothers, n** ***(%)***	
Low	2 *(15.4)*
Middle	3 *(23.1)*
High	8 *(61.5)*
**Education level partners, n** ***(%)***	
Low	1 *(7.7)*
Middle	4 *(30.8)*
High	8 *(61.5)*
**Ethnic background, n** ***(%)***	
Moroccan	9 *(69.2)*
Turkish	2 *(15.4)*
Dutch	1 *(7.7)*
Others	1 *(7.7)*
**Employment status, n** ***(%)***	
Not in paid employment	8 *(61.5)*
Part-time employment	4 *(30.8)*
Sickness leave	*1 (7.7)*
**Number of children, n** ***(%)***	
1	5 *(38.4)*
2	4 *(30.8)*
3	4 *(30.8)*
**Age of infant in months, mean** ***(range)***	15.9 *(1–24)*
**Age categories of infants, n (*%*)**	
0–3 months	1 *(5.9)*
3–6 months	1 *(5.9)*
6–12 months	6 *(35.3)*
12–24 months	9 *(52.9)*

An analysis of the interviews and observational log data identified five main themes that captured the experiences of the mothers in the parent-child meetings and how these meetings supported them in promoting a healthy lifestyle for their children: 1) the parent-child meetings were found to be enjoyable and contributed to feeling engaged with the neighbourhood; 2) being engaged in activities and playing together fostered parent-child interactions; 3) receiving social support and learning from other mothers were found to be important; 4) relevant information and learning in practice were key to making lifestyle changes, and involving children during workshops was essential; and 5) the positive attitude of health professionals and safe climate of the meeting were crucial in taking on their advice.

### The parent-child meetings were found to be enjoyable and contributed to feeling engaged with the neighbourhood

The main reason for mothers to attend the parent-child meetings was the opportunity for their children to meet other children, play together and socialize with other children, which they thought would benefit their socio-emotional, cognitive and motor development. The informal approach of the facilitator stimulated a relaxed and safe atmosphere for the mothers. The mothers also valued the city farm as a location, which enabled them to provide their children with the opportunity to come into contact with animals, nature and to play outside. Furthermore, the pleasure and fun they experienced in undertaking activities with other mothers was appreciated by all. Although the meetings were organized in a specific district, mothers from different neighbourhoods or cities also attended the parent-child meetings because they found them very enjoyable. Some mothers indicated that there were few opportunities in their neighbourhood to do something outside with their child(ren) or to take part in activities and meet other parents.

*‘A mother told me today: “Yes, it is just really fun for the children. There is nothing here for children of this age, I hear that a lot from other parents too, that they don’t know how… you want to entertain your children, you don’t want to keep them home all day. And here they really learn how to share and interact with other children. My child has older siblings, but that is not the same.”’ (From field notes)*

Some mothers stated that they felt isolated at home after their baby was born and remarked that the parent-child meetings were a reason for them to get out of the house and be involved in society again, and that they contributed to them feeling engaged with other people and the neighbourhood. The mothers also said that attending the parent-child meetings made them more aware of the activities in the neighbourhood, which contributed to them participating more in various activities that were organized in the neighbourhood.

*‘I was home for a long time after two pregnancies, and this is my first step towards participating in something social. So for me, it was quite a big thing, a big step to do something with other people, because I have had a very rough two years. So I think this is important for many parents who are taking their first steps in taking part in society again.’ (From interview)*

It was also noticeable that after a while some mothers gave more input about future activities and some of them also offered support in preparing and leading an activity. These mothers were very enthusiastic about the meetings and said that feeling engaged with the neighbourhood and activities encouraged them to think about other possible activities during the meetings and motivated them to contribute ideas.

*‘A mother asked me today: “I have been thinking about several activities since my visits here, which could be fun for the children and also for us. I would like to share with you these other activities we could do with the children. Would you like it if I prepared the activity for next week?”’ (From field notes)*

### Being engaged in activities and playing together fostered parent-child interactions

The set-up of the meetings included a lot of moments for the mothers to play and interact with their children. There were indications that the meetings fostered this parent-child interaction. After some time, it was observed that mothers played more with their children and became increasingly engaged in the activities during the meetings. For example, at the beginning, mothers would put their children on mats on the floor and sit on a chair, which did not invite interaction with their child, who was playing on the floor. After several meetings, the facilitators placed additional mats next to the chairs and this led the mothers to more often sit next to their child on the floor, which fostered their engagement in the activities and interaction with their child. Several mothers also remarked that they spent more time with their child at home since their visits to the parent-child meetings, and that they felt that this improved the relationship with their child. Some mothers expressed that before the parent-child meetings they were struggling to figure out how to play with their child, that they did not know what to do and were unsure about what activities were age appropriate. The various arts and crafts and physical activities during the meetings were a source of inspiration about how to play with their child and encouraged them to try this at home.

*‘Myself, I have two left hands, so I am not very good at drawing and clay modelling and that sort of thing. What this brought me is that I have started colouring, drawing, tinkering with her, even though I am not good at it. Because we do it here together, we now do it at home as well, I play and talk more with her.’ (From interview)*

Some mothers indicated that it was not common in their culture to communicate, interact or play actively with young children, and therefore they had a lack of knowledge and experience about how to entertain a child and spend time together.

*‘I think that a lot of mothers here learn how to play with their child. I did not know how to play with my child, because my mother never did that with me. It sounds very easy: “play with your child”, but if you have never seen or learned it, and have only seen the caring side... So that your mother feeds and washes you, but never plays with you, which is the case with many Moroccan mothers, then I won’t learn that either.’ (From interview)*

### Receiving social support and learning from other mothers were found to be important

Contact with other mothers was experienced as an important source of social and peer support because the women realized that other parents were experiencing similar challenges in raising a child and they could sympathize more easily with the situation. It was reassuring for them to hear that other parents experienced similar uncertainties, worries or problems. In addition, the mothers stressed that other parents at the meetings passed on useful strategies for dealing with challenging situations. Some mothers remarked, for example, that they preferred to talk with mothers at the parent-child meetings rather than family members or their closer network, since they did not always agree with the practices or beliefs of their family and therefore found it difficult to discuss some issues with them. The mothers experienced mutual understanding and felt that other mothers were impartial and would not judge them.

*‘It is easier here sometimes, when it concerns the extended family, the mother or the mother-in-law who is stuffing your child with all sorts of things, while you think that two sandwiches is enough because she is full. There are other mothers here who can relate to that and that makes it easier to talk.’ (From interview)*

Additionally, the parent-child meetings provided informal opportunities for the mothers to exchange practical information and tips with other mothers. This peer contact was highly valued by the mothers, since the advice and tips they received from other women were based on experience and were therefore easier to implement in their own lives.

*‘You find yourself among other new mothers, mothers in the same situation. And you always have the same sort of conversations. You can search the internet as well, but it is nicer to just talk to someone about something. And also, you give each other information, you get information from others which is usually directly applicable to yourself.’ (From interview)*

Some mothers also said that they had learned a lot by observing how other mothers feed, play, communicate or interact with their children. For example, one mother expressed that she had learned new ways of giving vegetables to her child by following the example of another mother.

*‘I always tried to give her chopped vegetables for the taste, but that did not always work. And she (a mother at one of the meetings, RED) brought a plate with hummus and carrots and cucumber. And because of her, I now make such a plate for my daughter as well. That way, she eats more vegetables.’ (From interview)*

Furthermore, almost all of the mothers found it useful to observe other children of similar ages because this helped them gain more insight into the children’s developmental stages, and thus more ideas about what to expect from their own child at the various stages.

*‘You see other children and compare your child with other children of similar ages, what they can do, and then you think to yourself: “Oh, I have not tried that yet.”’ (From interview)*

### Relevant information and learning in practice were key to making lifestyle changes, and involving children during workshops was essential

The more informative parts of the parent-child meetings that addressed several health-related behaviours such as oral hygiene, healthy food or promoting physical activity were found to be useful and educational by the mothers. These workshops discussed health-related behaviours, addressing the mothers’ need for practical information and knowledge about these topics. All of the mothers said that these meetings added to their knowledge about healthy behaviours and gave them guidance, skills or practical information to use at home. The practical nature, or the way in which the information was provided, made it possible to apply the information or knowledge in their home situation. Most mothers indicated that these workshops contributed to developing their parental practices, such as preparing and providing more healthy food or doing more physical activities with their children. For example, during an interactive food workshop they learned new recipes or got ideas about providing more fruit and vegetables to their child, which they applied at home. It was also observed that the information or advice in the workshop was shared with and distributed to other mothers or grandmothers in the neighbourhood. One mother said, for example, that she received recipes from the ‘Healthy snack workshop’ from a grandmother who attended the workshop and shared the recipes on WhatsApp.

*‘Preparing and tasting snacks was the most educational for me personally. I learned it there, because I never used to have healthy snacks at home. I had to learn. And this is very important because I was somebody who used to grab chocolate or chips very easily. It just came automatically. Most mothers just give a biscuit or sweets, but during the activities you see how easy it is to make something and that the children like it also. They really eat it, and that makes you more willing to do it yourself too.’* (*From interview)*

Addressing unfamiliar topics such as oral health/hygiene/brushing children’s teeth were also very much welcomed by the majority of the mothers. They explained that the oral hygiene of their child was a topic they normally did not really think or talk about much, compared to topics such as feeding or sleeping patterns and related problems. All of the mothers said it was valuable to learn from the dentist who attended the meeting that they needed to start with oral hygiene before the age of 2, and that they did not know this. Also, they were unaware of the importance of tooth brushing for developing healthy teeth. The mothers indicated that they made more effort to take care of their child’s oral health after the workshop with the dentist. Also, they used the practical tips that were given.

*‘That time with the dentist, for example, when I heard how important it is to brush the teeth, made me do it at home too. She really had informative tips and showed us which position to use when brushing the teeth. If that dentist hadn’t been there, I never would have got this information.’ (From interview)*

Although the mothers were positive about the informative aspects of the parent-child meetings, they did not want it to impact on the playtime or the social interaction of the children and their mothers. Workshops that were designed to discuss health-related behaviours in an interactive way with mothers were found to be informative but less enjoyable for the children. For example, some mothers said that the workshop about healthy sleeping was very informative and interesting for them, but was not entertaining for their children. It was therefore difficult for them to follow the workshop, since it demanded too much concentration to take part in the discussion, while they were also interacting with their children. The workshops that were designed in an interactive way for both mother and child, in which they could learn skills or obtain information by doing it themselves, were highly valued and praised by mothers.

*‘A mother told me today: “Look, this is really fun, because we learn how to make healthy snacks, and she can join us because she can decorate something. But last week we had a lady here talking about sleep. Well, it might be good to discuss these sort of things, but it should not affect the playtime of the children. I come here to entertain my child, and that makes it difficult to join a discussion and keep your child busy at the same time. That child is going to be bored.”’ (Informal talk with a mother, from observation log date)*

### Positive attitude of health professionals and safe climate were crucial in taking on advice

The positive attitude of the health professionals who were involved in the parent-child meetings seemed to be crucial in making contact with the mothers. Being approachable and sharing real-life and practical experiences helped foster conversations on health and lifestyle themes and were better than giving a theoretical talk. For example, the workshop on oral health was praised by mothers because the dentist shared good and bad experiences with her own children and translated theoretical information into practical examples by sharing her own best practices. She also demonstrated to mothers individually how to take care of their child’s oral health. The majority of the mothers felt comfortable and safe to consult the health professionals or experts about their experiences and to ask questions. The mothers explained that the setting felt non-judgmental due to the informal set-up and presence of other mothers, and safer because they were not sitting alone with a professional in a room as they would in an individual consultation. Some mothers remarked that the presence of other parents made them feel less insecure and they were encouraged to ask the health professional questions because other mothers did so.

*‘I would not have gone to a practice like that myself, but on these mornings you are together with other mothers and not alone.’ (From interview)*

However, the workshops in which the health professionals did not provide much information or had a more passive attitude were less positively evaluated by the mothers. They remarked that they would have appreciated it if the health professional had guided them in the activities in the workshop and provided them with more general information about the benefits of the activity for their child’s development. Some mothers expressed that they did not know that the workshop was led by a health professional and therefore missed the opportunity to ask questions or get practical tips or guidance.

*‘That is a pity, we would have thought: “oh, an expert is watching too”. You know, as parents we could have asked her questions afterwards. Or she could have provided some information to us on what she’d seen.’ (From interview)*

## Discussion

In this study, we explored the experiences of parents after their participation in parent-child meetings and investigated *whether* and *how* these meetings supported them in providing a healthy lifestyle for their child. This study showed that the parent-child meetings were highly valued by the mothers, who indicated that the meetings contributed to improved parental knowledge, skills and practices regarding healthy behaviours of their children. The meetings helped them to manage the challenges associated with parenting, giving them the ability to stimulate healthy behaviour in their children by providing them with practical and relevant information that was demonstrated and practised during workshops. In particular, the peer support of other mothers, hands-on workshops and the provision of concrete advice and information in a non-judgmental and informal setting were highly valued by the mothers and mentioned as critical elements in adopting the new knowledge, skills and practices at home.

Many studies have shown that parent support groups help in the formation of friendships and social networks and in turn lead to an increased feeling of social connectedness with the neighbourhood and decreased feelings of social isolation [33–36, 49–51]. Mothers in our study also reported that the parent-child meetings contributed to feeling more engaged and involved with other people and activities in the neighbourhood, which resulted in feeling less isolated. Social connectedness is recognized as a basic human need and reflects the need for making meaningful connections and relationships with individuals, groups and/or institutions in the community, which posit a shared sense of belonging and generate opportunities for social support. Transition into parenthood challenges social connectedness and increases mothers risk becoming more socially isolated, because of feeling and being (physically) restricted due to extended periods of breastfeeding and birth-related health complications and thus have reduced and/or limited opportunities to interact with others [52]. It is well known that a lack of social connectedness and social support could increase the risk for (postnatal) depression and anxiety [53–56]. It is also well known that discussing parenting challenges with peers is reassuring for parents, because it provides them with a sense of empathy or being understood by individuals who are going through the same experiences and therefore fosters mutual understanding [31, 34, 50, 51]. Our study also found that the supportive climate was reassuring for many mothers. Sharing experiential stories with other mothers gave them the feeling that they were not alone in experiencing challenges with parenting.

Several studies have reported that parent groups could play an important role in providing social support, learning about parenting and increasing parental knowledge [57–59]. Mothers in our study also reported that the parent-child meetings increased their knowledge, confidence and efficacy in parenting skills and provided relevant information from peers. These aspects have also been identified in other studies [31, 34, 49, 50, 60, 61]. The presence of other parents also enabled them to observe and learn from others’ experiences or struggles. The importance of social support in peer learning and being more confident and growing as parent were also highlighted in other studies [42, 49, 62–64]. While the parent-child meetings were deductively developed and based on the findings from our earlier explorative study to the needs and experiences of parents in the first 2 years of their child’s life, it could be valuable to reflect on our findings by using the constructs of the social learning and cognitive theory [65, 66]. The social learning and cognitive theory posit that learning take place in a social context with a dynamic and reciprocal interaction of the person, environment and behaviour. It describes also that individuals can learn within a social context that is facilitated through modelling and observational learning and that they can adopt a behaviour successfully if they have seen a successful demonstration of it [65]. This will in turn increase the ability of someone to perform a behaviour, since they have gained knowledge and skills and know what and how to do a specific behaviour [66]. The enhanced self-efficacy may increase the confidence of one’s parenting skills and thus one’s ability to make health behaviour changes [66]. The design of the parent-child meetings were to provide support to parents through their experiences and sharing practical information to promote healthy behaviour in their children. Along with this, parent-child meetings focused building parenting competency and confidence regarding multiple health-related behaviours and were designed to create the conditions for change and to support families to adopt healthier behaviours by emphasizing on the provided information as well as on the delivery of the information. This would also contribute in building trustful relationship between (health) professionals and parents, which would make parents more receptive in taking advice regarding health-related behaviours and adopt it in their daily life [36, 39–41].

Most playgroups in other studies or districts have focused more on parenting practices that aim to stimulate the social and cognitive development of children, with an emphasis on educational outcomes (e.g. singing songs and rhymes, playing with colours, letters and numbers), rather than promoting health-related behaviours [59]. This also applies to playgroups in the Netherlands, which offer structured and unstructured play experiences for children and provide opportunities for parents and their children to connect and socialize, but focus less on healthy behaviours. While this structure and the relaxed setting of playgroups provide a unique opportunity to support parents in their practices regarding health-related behaviours, there are limited interventions targeting such behaviour in these settings and most of them focus on promoting physical activity or nutrition [35, 38, 67, 68]. A previous study among parents in Australia identified barriers to and facilitators of the development of a potential childhood obesity prevention programme, with a broader focus on lifestyle domains which could be delivered in community playgroups [31]. They found that parents were enthusiastic about a potential programme that focused on lifestyle factors, but they did not want any formal programme to encroach on the ‘playgroup time’ of their children and the relaxed atmosphere of the playgroup [31]. Mothers in our study confirmed these findings by underlining the importance of learning about health-related behaviours while they have a fun and enjoyable time with their children.

Following on from this point, the aim of prevention programmes is to intervene before problems arise and it is argued that these kinds of programmes have the tendency to focus on negative aspects, which could create a feeling of insufficiency regarding parenting and could negatively influence parents [69]. In addition to this assumption, our study provided insights into *how* the programmes that are delivered in such circumstances could be facilitated, by taking a supportive approach rather than a problem-centred approach. We found that mothers positively evaluated the educational and informational component regarding health-related behaviours when it was provided in an interactive way, involving both the children and the parents in the activities. For example, demonstrative workshops such as ‘oral health/teeth brushing’ or ‘making healthy snacks’ involved parents and children in the activities and provided parents with pragmatic, realistic and applicable information without feeling they were being ‘taught’ about parenting. This is an important point, since it is argued that a lot of parent support programmes are designed from a ‘professional point of view’ and delivered within the prevailing ‘pedagogicalized’ climate, in which parents are taught how to parent [69]. However, a growing body of evidence shows that parents want information from trusted sources, including both health professionals and peers, in an observational and socially-constructed learning environment [21, 31, 36, 60, 69].

In addition, our data showed that the attitude of the health professional was crucial in creating a supportive climate and making contact with the mothers. We found that mothers more readily took the advice of health professionals when the latter shared their own real-life and practical experiences as a parent, which minimized the distance between the mothers and the professionals, putting them all on the same level. Communicating with professionals in a community setting was more low-key and relaxed for the mothers, compared to a one-to-one consultation in a formal therapeutic setting (e.g. office of paediatrician or dentist). The mothers reported, for example, that they felt more comfortable in the parent-child meetings because the presence of other parents gave it a less formal, community structure.

Furthermore, some mothers expressed the view that the parent-child meetings were a stimulus for changing their parenting beliefs and that they learned new perspectives and skills in child rearing which improved their interaction with their child. For example, some mothers said that they had not learnt to interact or communicate verbally with their children through their own upbringing, as they had only seen the physical and practical caring side of parenting in their own mothers (e.g. feeding, bathing). These mothers were less able to play or construct a relationship with their children in the first few years of their children’s lives. Indeed, it has been described that parents need to have experienced nurturing relationships themselves in order to provide the same to their children [70]. Because parenting differences depend on a variety of factors, including cultural and family traditions, life experiences, interpersonal relationships and individual subjectivity [71–75], it might be interesting to further explore how these factors play a role in shaping parenting in terms of health-related behaviours.

### Strengths and limitations

An important strength of this study is the combination of approaches to data collection, which included participatory observations, informal conversations and formal interviews. The use of participatory observation and informal conversations allowed us to become acquainted with the participants in an informal way, and provided opportunities to explore and understand social interactions and behaviours that occurred within a group of people or culture [76, 77]. This enabled us to gain insights into participants’ actions and perspectives in their natural setting, which could not be obtained through formal interviews. The participatory observations also contributed to building positive relationships with participants and made it easier to approach mothers for the formal interviews. These interviews with participants complemented the observations and provided additional insights into the experiences and perspectives of participants with regard to activities during the parent-child meetings, and resulted in richer and more detailed data, including insights into their behaviours in the home setting.

One of the limitations of the present study is the small number of participating fathers and other caregivers such as grandparents or aunts. It is known that fathers and grandparents exert a considerable influence on children’s behaviour and lifestyle [28, 78–82]. By obtaining greater insight into the needs of both fathers and grandparents, and making future parent-child meetings more attractive to them, it will be possible to obtain insight into their experiences.

Furthermore, the parent-child meetings were organized in a neighbourhood inhabited by culturally and linguistically diverse families. The majority of the participants were able to understand and communicate in Dutch, but there were also parents and grandparents in attendance who did not speak or understand Dutch. This could have an influence on the levels of consistent attendance and active engagement of both parents and grandparents who do not speak Dutch. The availability of a bilingual facilitator is therefore valuable and recommended, since it has been shown that language could be an important factor in the attendance and engagement level of participants [36]. It would also be helpful if the activities were also suited to participants who are less proficient in the predominant language, in this case, Dutch.

### Recommendations for policy and practice

We identified some key elements that can be used for the development of a successful parenting programme that aims to promote healthy behaviour in the early years. First of all, the results of our study and the literature show that it is important to use a more participatory approach and engage parents in the development of the parenting programmes [41]. An active collaboration with parents from the beginning will contribute in developing a health promotion program that fits within the needs of the target group and will increase the sense of owner- and partnership [36, 39, 42]. Our results also showed that it is important to organize these programs in an informal, accessible and safe environment, since this enabled facilitators and parents to develop relationships based on trust and respect. Practical suggestions for facilitators and professionals to facilitate this include the use a non-judgemental approach and to provide health-related information in combination with fun and interactive practical activities for both mothers and their children. Furthermore, it is important that these programs are accessible for parents from different socioeconomic backgrounds, and therefore we suggest that access is free of charge or for a small contribution, so that all families and children are given the opportunity to attend and learn about health-related behaviours. This could potentially lead to the reduction of socioeconomic inequalities in health behaviours.

## Conclusion

This study showed that participating in a parent support group, the ‘Parent-Child Meetings’, contributed to enhanced knowledge, skills and practices of parents regarding healthy behaviours of their children. This could potentially benefit the health of children during the first 2 years of their lives. In particular, the peer support of other parents, the hands-on workshops and the concrete advice and information provided in an informal and non-judgmental setting were highly valued by the parents.

Future parent support groups could use these results to improve their meetings or to start meetings that better suit the needs of parents with children aged 0–2 years. Finally, further research is needed to investigate whether the improvements in parental knowledge, skills and practices concerning a healthy lifestyle for their children ultimately lead to the better health of the children.

## Supplementary Information

**Additional file 1:.** Interview guide on the experiences of mothers in the parent-child meetings.

## Data Availability

The qualitative datasets generated and/or analysed during the current study are not publicly available due to the data containing information that could compromise research participant privacy/consent, but are available from the principal researcher G. Bektas (g.bektas@vu.nl) on reasonable request, and subject to approval from the research committee of the Vrije University Medical Centre Amsterdam.
