# Correction to: Supporting parents and healthy behaviours through parent-child meetings – a qualitative study in the Netherlands

**DOI:** 10.1186/s12889-021-11440-1

**Published:** 2021-07-12

**Authors:** Gülcan Bektas, Femke Boelsma, Carline L. Wesdorp, Jacob C. Seidell, Vivianne E. Baur, S. Coosje Dijkstra

**Affiliations:** 1 Department of Health Sciences, Faculty of Science, Vrije Universiteit Amsterdam, Amsterdam Public Health Research Institute, Van der Boechorstraat 7, 1081 BT Amsterdam, the Netherlands; 2 Department of Care Ethics, University of Humanistic Studies, Kromme Nieuwegracht 29, 3512 HD Utrecht, the Netherlands

**Correction to: BMC Public Health 21, 1169 (2021)**

**DOI: 10.1186/s12889-021-11248-z**

It was highlighted that in the original article [1] and specifically in the Background section the sentence “However, most interventions in playgroup settings focus on promoting one healthy behavior, such as healthy food or physical activity, and there are limited studies in these kinds of settings that target multiple health-related behavior domain together” was erroneously duplicated. The original article has been updated.
