# Supplementary material for: Supporting parents and healthy behaviours through parent-child meetings – a qualitative study in the Netherlands
Source: BMC Public Health. 2021 Jun 18;21:1169. doi: 10.1186/s12889-021-11248-z (PMC8211718; doi:10.1186/s12889-021-11248-z)
Supplement: Supplementary file 1 — Additional file 1:. Interview guide on the experiences of mothers in the parent-child meetings. [file 12889_2021_11248_MOESM1_ESM.docx]

**Supplementary material**

**Appendix A. Interview guide on the experiences of mothers in the parent-child meetings**

- Why did you attend the parent-child meetings? What was the reason for your attendance (what attracted you most)?
- What do you think of the activities during the parent-child meetings?
- What did the parent-child meetings offer you?
- What did you learn from the activities during the parent-child meetings?
- Which elements or aspects did you adopt in your home setting or try out at home?
- What did the parent-child meetings offer your child(ren)?
- Which activity did you like the most?
- What do you think about providing information regarding healthy behaviour during the meetings?
- Which activity was most informative and/or useful and why?
- What do you think about meeting other parents with children in the same age group during the parent-child meetings?
- How important do you think it is to have a network of parents with children in the same age group?
- Did the parent-child meetings contribute to or expand your network of parents?
- Did you already know parents at the meetings?
- Is it easier to come to the meetings if you know that there will be other parents who you already know?
- Have you also met new parents?
- Do you talk to or have conversations with the parents at the meetings?
- Which topics do you talk about?
- Do you also talk about problems or tips about promoting a healthy lifestyle for your child?
- Did the parent-child meetings contribute to a connectedness with the neighbourhood or activities? How did it contribute or change your feelings of connectedness?
- Would you recommend the parent-child meetings to other parents, and why?
- What else should be offered in the meetings?
- What could be improved or what did you find missing from the meetings?
